# Sir William Broadbent on Consumption

**Published:** 1906-02-10

**Authors:** 


### The Hospital.

A Journal of

The Medical Sciences and Hospital Administration.

Vol. XXXIX.—No. 1,011. SATURDAY, FEBRUARY 10, 1906.

### SIR WILLIAM BROADBENT ON CONSUMPTION.

Sir William Broadbent's memorial to the Metropolitan Asylums Board, urging them to become the local sanitary authority for tuberculosis, and in that capacity to arrange for the notification of pulmonary consumption, and more especially for reception in one or more of their hospitals of the many incurable cases which are now distributing their bacilli among the community, is likely to attract a considerable degree of public attention; and the determination at which the Board will arrive will be expected with much interest by large sections of the community. It may be presumed that the question will require more prolonged consideration than that of a single meeting, and it is more than probable that some special committee of the Board may be appointed to inquire and to report as a preliminary to action. Sir William speaks of the disease as "costing the country tens of thousands of lives and millions of money every year," and it is worth while to look at the facts of its prevalence in the light thrown upon them by the last report of Sir Shirley Murphy to the London County Council, which deals with the statistics of the year 1904, and with the "administrative county" of London, not including the City. The mortality in the county from pulmonary phthisis in successive periods, from 1850 to the present day, has steadily decreased from 2.86 per thousand persons living in 1850 to 1.55 per thousand living in 1903; but in 1904 it rose to 1.62, a point intermediate between the 1.66 of 1901 and the 1.60 of 1902. The actual numbers of deaths were 7,526 in 1904, as compared with 7,124 in 1903. Sir Shirley makes no attempt to explain the 1904 increase of mortality, but he calls attention to the marked general decline, and refers to the factor of uncertainty arising from the more exact diagnoses of recent years. Accepting the registration returns as accurate, he observes that the London death-rate from phthisis at all ages has with but slight exceptions been greater than that of England and Wales; and he shows that the phthisis death-rates per thousand living in England and Wales, for the decennium 1891-1900, were, males 1.574, females 1.209; while the same figures for London were, males 2.302, females 1.355. Taking the phthisis death-rates of all ages of the decennium 1851-60 as 1,000, the rates for England and Wales of the decennium 1891-1900 were, males 610, females 436; and those for London were, males 700, females 544. It is manifest that the disease is far more fatal in the metropolis than elsewhere, although the real extent of the evil in the metropolis is unquestionably concealed by the fact that numbers of persons who contract phthisis in the course of London occupations return to their provincial homes to die amongst their own people. Every country practitioner is familiar with examples.

The measures at present adopted for the control of phthisis in London are of a very tentative and imperfect description, and it is manifest that the declining prevalence of the disease must be attributed rather to the effects of general sanitary improvements than to any special efforts made in relation to it. The notification, which Sir William Broadbent desires to see general, is at present only voluntary and partial, and in the year 1904 was in operation in 18 districts out of 29. Since then it may have been adopted in others; but in the report from which we quote the districts of Paddington, Hackney, Shoreditch, Bethnal Green, Stepney, Poplar, Battersea, Camberwell, Deptford, and Lewisham were still content to remain ignorant of the amount of phthisis existing within their several boundaries. St. Pancras, we understand, commenced a system of voluntary notification with the present year. The authorities of Stoke Newington and of Finsbury express the opinion that much of the value of the notifications is lost owing to the fact that notice is seldom given until the disease has reached an advanced stage; and, in some of the reports, the need for compulsory notification is strongly insisted upon. In the London districts in which voluntary notification is now encouraged, the sufferers are visited and advised as to the precautions which should be taken in their homes, and, when not objected to, disinfection of the premises follows removal or death. Sir William Broadbent's memorial, which was published in the *Times* of the 2nd instant, urges upon the Asylums Board not only that notification should be at once enforced for all patients with phthisis, falling under the observation of Poor Law medical officers or public institutions, but that one or more of the hospitals of the Board should be appropriated to their reception, and that they should be removed from their homes whenever possible in order to diminish the danger of infection. The County Council has made a by-law forbidding expectoration on the floors and walls of public conveyances, public halls, waiting-rooms, and places of entertainment; and efforts are being made to teach phthisical patients to expectorate into suitable receptacles; but it is impossible to walk along the London streets without observing evidence of the small degree of success with which these efforts have so far been attended. We once saw in a public hall in America a distribution of receptacles on the floor and a series of wall notices: "Gentlemen *will*, and others *must*, use the spittoons." Unfortunately the "gentlemen" would not, and the "others" did not; and much the same conditions exist among ourselves.

